# Lifestyle Factors in Cancer Survivorship: Where We Are and Where We Are Headed

**DOI:** 10.3390/jpm5030243

**Published:** 2015-07-02

**Authors:** Namrata Vijayvergia, Crystal S. Denlinger

**Affiliations:** Department of Medical Oncology, Fox Chase Cancer Center, 333 Cottman Ave, Philadelphia, PA 19111, USA; E-Mail: namrata.vijayvergia@fccc.edu

**Keywords:** lifestyle factors, cancer survivorship, physical activity, dietary factors, smoking

## Abstract

Advances in early detection and curative therapies have led to an increased number of cancer survivors over the last twenty years. With this population comes the need to evaluate the late and long term effects of cancer treatment and develop recommendations about how to optimally care for these survivors. Lifestyle factors (diet, body weight, physical activity, and smoking) have been linked to a higher risk of many medical comorbidities (cardiovascular, metabolic, *etc.*). There is increasing evidence linking these factors to the risk of developing cancer and likely cancer-related outcomes. This link has been studied extensively in common cancers like breast, colon, prostate, and lung cancers through observational studies and is now being prospectively evaluated in interventional studies. Realizing that survivors are highly motivated to improve their overall health after a diagnosis of cancer, healthy lifestyle recommendations from oncology providers can serve as a strong tool to motivate survivors to adopt health behavior changes. Our article aims to review the evidence that links lifestyle factors to cancer outcomes and provides clinical recommendations for cancer survivors.

## 1. Introduction

A survivor is defined as a person diagnosed with cancer “from the time of diagnosis and for the balance of life”; this definition also includes family, friends, and caregivers [1]. Advances in early detection and curative therapies, coupled with increased life expectancy and growth of an aging population, has led to an increase in the number of Americans with a history of cancer. Nearly 14.5 million Americans were cancer survivors in 2014, and 64% of them are long term survivors (diagnosed more than five years ago) [2]. It is estimated that by January 2024, the population of cancer survivors will increase to almost 19 million [2].

Cancer survivors, including long-term survivors, have poorer health outcomes than do similar individuals without cancer, independent of the time since diagnosis [3]. In addition to risks for recurrence and the development of second primary malignancies, cancer survivors have a higher mortality due to non-cancer causes, most commonly cardio-respiratory diseases [4,5,6,7]. In fact, coronary artery disease becomes the leading cause of death in breast, endometrial, and colorectal cancer survivors approximately ten years after diagnosis [8,9,10]. Mediastinal radiotherapy and systemic chemotherapy can also induce late cardiovascular adverse effects like myocardial infarction and cardiac insufficiency [11,12]. Cancer survivors are also more likely that those in the general population to develop a second primary cancer, as lifestyle behaviors like smoking and alcohol intake account for 35% of excess cancers in this population. Similarly, other lifestyle risk factors (e.g., excess body weight, physical activity) also contribute to the increased risk of second primary malignancies [13].

Survivorship care focuses on health and well-being after treatment and encompasses physical, psychological, and socio-economic issues. There are four essential elements of survivorship care as defined by the Institute of Medicine: surveillance for recurrent cancer, new primary cancers, and medical and psychosocial late effects of cancer and its treatment; prevention of recurrent or new cancers and of late effects of treatment; intervention for consequences of cancer and its treatment; and coordination of care between oncology specialists and primary care physicians [14]. Attention to all facets of care is important to ensure that cancer survivors receive comprehensive survivorship care.

Given the increasing number of long-term survivors, and the possibility of competing causes of morbidity and mortality in this population, there is an increasing amount of literature addressing the role of lifestyle behaviors in the survivor population. Epidemiological and interventional studies evaluating the engagement and promotion of healthy eating, weight control, and physical activity have been published, suggesting that lifestyle behaviors may be important to counter some of the adverse effects of cancer treatments and disease recurrence while improving overall health outcomes [15]. Lifestyle interventions are important aspects of survivorship care, as cohort studies have suggested that engagement in physical activity or adherence to a healthy diet may impact overall quality of life as well as disease-specific and overall health outcomes in certain tumor types. We review the impact of healthy lifestyle behaviors in cancer survivors. The summary of recommendations is tabulated in the Appendix.

## 2. Weight Management in Cancer Survivors

Excess body weight is a risk factor for multiple cancers, with greatest associations in breast, colorectal, prostate, esophagus, and pancreatic cancers [16,17]. Obesity may also be associated with an increased risk of cancers of the liver, cervix, and ovary, as well as non-Hodgkin lymphoma, multiple myeloma, and aggressive forms of prostate cancer [18]. Various molecular mechanisms have been put forth to explain the association between body weight and cancer risk, including the impact of low-grade chronic inflammation, elevated levels of leptin and adiponectin, altered levels of hormones and growth factors (insulin, insulin-like growth factor-1, estrogens, and androgens), insulin resistance, and PI3K-AKT-mTOR signaling pathway alterations [16,19]. Unfortunately, an increasing number of patients who are overweight or obese at the start of cancer therapy gain additional weight as a complication of treatment [15,20]. It is estimated that approximately 70% of breast and prostate cancer survivors are overweight or obese [21]. This trend toward increased obesity in cancer survivors may impact cancer-related outcomes, as a prospectively studied cohort of over 900,000 healthy adults demonstrated that a BMI ≥ 40 kg/m^2^ was associated with 52% and 62% higher rate of cancer-related mortality in men and women, respectively [17].

The impact of post-treatment weight gain has been studied most extensively in women with breast cancer, where weight gain is associated with higher cancer-specific and all-cause mortality [22,23,24]. A meta-analysis of over 200,000 breast cancer patients demonstrated a 75% and 34% increase in mortality among obese pre- and postmenopausal women, respectively, as compared with patients who were of normal weight at diagnosis [25]. Potential causes of this increased mortality include the practice of chemotherapy dose reductions due to the use of adjusted body weight or the selection of less aggressive therapy in obese individuals, which may confound results of observational studies. However, data from clinical trials, where dosing is mandated to be based on actual body surface area, confirm this increased mortality. In the adjuvant chemotherapy therapy E1199 study, a BMI ≥ 30 kg/m^2^ was associated with poorer prognosis in women with hormone-receptor positive tumors, compared to women with a normal BMI [26]. Similar analysis of the ATAC study (adjuvant endocrine therapy in postmenopausal women hormone receptor–positive, stage I to III breast cancer) showed that women who had a BMI of 35 kg/m^2^ or higher had an increased risk of recurrence compared to women with a BMI less than 23 kg/m^2^ [27].

In colorectal and prostate cancer, available evidence suggests an unfavorable effect of increased adiposity on survival, although some inconsistencies exist in the link between obesity and cancer outcomes [28]. A cohort study of over 4000 patients with colon cancer suggested that patients with a BMI >35 kg/m^2^ at baseline had greater risk of colon cancer recurrence or second primary malignancies when compared to normal weight patients [29]. Two adjuvant colorectal cancer studies have shown a higher risk of colorectal cancer recurrence, cancer-specific and all-cause mortality in obese survivors [30,31]. However, a study of stage III colon cancer patients treated with adjuvant fluoropyrimidine-based chemotherapy failed to confirm an association between BMI and cancer-specific outcomes [32]. A recent meta-analysis of 22 studies evaluating prostate cancer and obesity demonstrated a 20% increase in risk of prostate cancer-specific mortality and a 21% increase in risk of biochemical recurrence for each 5 kg/m^2^ increase in BMI above normal [33]. Obesity has also been associated with an increased risk of biologically aggressive prostate cancer and more advanced disease at the time of diagnosis [34,35].

Much less evidence exists regarding the relationship between weight change after diagnosis and outcomes. The Women’s Intervention Nutrition Study (WINS) found that a low-fat diet causing a 6 lb. weight loss reduced the risk of recurrence among postmenopausal breast cancer survivors by 24%, with greatest impact in those survivors with hormone receptor negative tumors [36]. However, results were confounded by the impact of a low-fat diet alone on these outcomes. Initial results of the LISA (Lifestyle Intervention Study for Adjuvant Treatment of Early Breast Cancer) trial, in which 338 postmenopausal women with hormone receptor-positive breast cancer were randomly assigned to an educational control group or a two-year telephone-based weight-loss intervention, suggested that intervention participants lost approximately 4.5 kg more and reported an improvement in physical functioning compared with control group. Cancer-specific outcomes are still pending [37]. Randomized trials have not evaluated the impact of purposeful weight loss on cancer outcomes in colon or prostate cancer. The few published observational studies did not show benefit of weight loss after diagnosis in colon cancer. For example, data from an adjuvant colon cancer trial studying fluoropyrimidine-based therapy in stage III disease (CALBG 89803) and another observational study failed to demonstrate a significant association between weight change after diagnosis and risk of cancer recurrence and death [32,38].

Efforts are underway to more formally determine whether structured weight loss interventions can improve outcomes in certain cancer types. The Exercise and Nutrition to Enhance Recovery and Good Health for You (ENERGY) study is designed to study over 700 women with early stage breast cancer to demonstrate the feasibility and impact of weight loss on quality of life and other cancer related outcomes [39]. A second study, the Women in Steady Exercise Research (WISER) study, is evaluating weight loss programs with or without structured physical activity in breast cancer survivors as well [40]. In colorectal cancer, internet-based and behavioral weight loss interventions are being evaluated in survivors. Weight loss programs and other interventions are also being evaluated in prostate cancer, endometrial, and childhood cancer survivors [41].

Despite the conflicting data on weight, weight loss, and cancer-specific outcomes, the goal for all survivors should be to achieve and maintain a healthy body weight (BMI 18.5–25 kg/m^2^) in order to maximize overall health outcomes. The American Cancer Society (ACS) has developed guidelines for cancer survivors, which include a recommendation to achieve and maintain a healthy weight throughout the balance of a survivor’s life [42]. In most survivors, needed intentional weight loss efforts can be deferred until cancer therapy is complete. Weight loss of even 5% to 10% is likely to have significant health and cardiovascular benefits [43]. If cancer survivors are overweight and motivated to pursue weight loss, losing a maximum of two pounds per week in a healthy fashion is acceptable and encouraged as long it is monitored and it does not interfere with treatment [44,45]. After cancer treatment, weight gain or loss should be managed with a combination of dietary, physical activity, and behavioral strategies. This should be achieved through limitation in the consumption of high-calorie foods and beverages and increased physical activity [18]. Limiting portion sizes, especially of energy-dense foods, is an important weight loss strategy [46]. As many oncology clinicians are not optimally trained in weight loss strategies, the use of available resources and tools for weight loss counseling is recommended. For example, the American Society of Clinical Oncology (ASCO) recently developed a tool for clinicians to manage obesity that involves education and awareness about energy balance and cancer risk association; and provides tools, guidelines and resources to help providers and survivors tackle obesity. ASCO also included a position statement on obesity, reinforcing the need to promote research on obesity cancer links and formulate policies to improve access to evidence-based obesity treatment services for cancer patients and survivors [47]. The National Comprehensive Cancer Network (NCCN) Survivorship Guidelines have incorporated guidelines on weight management in survivors [48] The American Institute for Cancer Research (AICR) has also developed patient-friendly materials addressing weight management and weight loss strategies [42].

## 3. Diet and Dietary Supplements in Survivors

A number of cohort studies have evaluated survivors’ dietary habits and the impact of diet on cancer-related outcomes and overall mortality. Akin to outcomes in the general population, increased fat and energy intake has been associated with lower risk of recurrence and death in cancer survivors [45,49,50,51]. An observational study of Nurses’ Health Study participants who were diagnosed with invasive breast cancer suggested that consuming a healthy diet (high in fruits, vegetables, whole grains) may or may not lower cancer-specific mortality but significantly lowers mortality from other disease (15% reduction in all-cause mortality) as compared to a typical western diet [22]. Another cohort study of self-reported dietary habits in breast cancer survivors suggested that a combination of healthy diet (five servings per day of fruits and vegetables) and physical activity (equivalent to walking 30 min 6 days per week) was associated with a 50% reduction in mortality over a seven-year follow-up period [52]. A low-fat diet causing a 6 lb. weight loss reduced the risk of recurrence among postmenopausal breast cancer survivors by 24% in the WINS study, although it is unclear as to which component (low fat diet or weight loss) contributed greater [36]. On the contrary, the Women’s Healthy Eating and Living (WHEL) study of breast cancer survivors did not show a difference in recurrence-free survival between the experimental (diet low in fat and very high in vegetables, fruits, and fiber) and the control arms. Of note, women in the WHEL study intervention group did not exhibit weight loss, suggesting that diet alone may not be enough to affect cancer-specific outcomes [53].

The role of diet content on outcomes has been studied in colon cancer as well. Evaluation of dietary habits in CALGB 89803 participants found that higher intake of a Western diet (refined/processed food with high intake of fat and red meat) was associated with significantly higher risk of recurrence and mortality [54]. Meyerhardt *et al.* also found that glycemic load impacted disease free and overall survival in stage III colon cancer patients, with higher dietary glycemic load significantly associated with worse disease-free survival among overweight or obese colon cancer survivors (BMI ≥25 kg/m^2^) [55]. In prostate cancer, saturated fat intake (but not total fat) was associated with worse survival, while monounsaturated fat intake was associated with improved outcomes [56,57]. Another study showed a significant decrease in PSA in advanced prostate cancer patients randomly assigned to a low-fat, vegan diet group *versus* controls (6% *vs.* 4%; *p* = 0.016, n = 93) [58].

Despite the demonstrated benefit of healthy eating habits in cancer survivors, studies suggest that approximately 50%–70% of survivors do not meet the current dietary guidelines. The ACS-SCS II study surveyed over 9000 survivors across six different cancer types and suggested that less than 20% of cancer survivors reported meeting the 5-A-Day dietary recommendation (five servings of fruits and vegetables per day) [59]. A major barrier to healthy eating is lack of information, as physicians often do not discuss dietary habits with survivors during the clinic visit. Only 10% of cancer survivors report being asked or advised about diet and exercise [60]. Results from our own work suggest that despite educating oncologists on the importance of healthy diet for survivors, only 14% of colorectal cancer and 22% of lung cancer survivors had documentation of healthy diet counseling from their providers. Lack of time during the clinic visit was reported to be a major limitation in implementation of these practices [61].

The role of dietary supplements after cancer treatment is also of interest to many survivors. Dietary supplement use is reported by 64%–81% of cancer survivors [62,63], with 15%–30% of cancer survivors initiating supplement use after their cancer diagnosis [62]. However, the benefit of such supplements is questionable. Use of multivitamins did not improve recurrence risk, cancer-specific or overall mortality among women diagnosed with early stage breast cancer [64,65]. This observation held true for colon cancer survivors as well [66]. A large Finnish study found no reduction in the incidence of lung cancer among male smokers after prolonged dietary supplementation with vitamin E or beta carotene. In fact, they observed an 18% higher incidence of lung cancer in patients receiving beta carotene [67]. Similarly, the Selenium and Vitamin E Cancer Prevention Trial (SELECT) found that men receiving supplemental selenium or vitamin E had a higher incidence of diabetes and prostate cancer, respectively [68]. Two colorectal cancer-specific studies and a systematic review of several studies involving a variety of cancer types found a reciprocal prognostic role for low serum vitamin D level in patients with many cancer subtypes. Unfortunately, the WHEL, Women’s Health Initiative study and a meta-analysis of three prostate cancer-specific studies failed to demonstrate a benefit of vitamin D supplementation on cancer-related outcomes [69,70,71,72,73,74].

With increasing knowledge about the importance of healthier habits to prevent cancer, survivors are motivated to improve their lifestyle habits after diagnosis, including improvements in diet [75]. Nutritional assessment should be an integral part of the treatment plan for cancer survivors, beginning at diagnosis and extending throughout the post-treatment period [76]. The ACS, NCCN, and AICR [42,48,77] have all published guidelines or recommendations on nutrition and diet specifically for cancer survivors, with recommendations that survivors strive to meet their nutritional needs through food rather than supplements. Providers should counsel survivors to achieve a dietary pattern that is high in vegetables, fruits, and whole grains, and consume at least 2.5 cups of fruits and vegetables a day [18]. NCCN also recommends that providers assess survivors’ daily intake of fruits and vegetables and counsel survivors to limit foods with added fat/sugar, processed foods, red meats and alcohol. Evaluation of portion sizes, night grazing and eating out patterns should be done as well. Both AICR and NCCN recommend a diet rich in vegetables, fruits and whole grains (2/3^rd^ of the plate), with less emphasis on animal proteins (1/3rd part of the plate). Recommended sources of dietary fat include plant sources (olive/canola oil, avocados) and fatty fish, rather than red meats. Both the ACS and NCCN recommend against the routine use of dietary supplements for most survivors, unless supplementation is indicated for a documented dietary deficiency or issue with nutrient absorption [18,48]. ACS endorses the practice of daily multivitamin use during and after cancer therapy for those who are unable to meet their nutritional needs through diet alone. In addition, given the lack of a documented cancer control benefit with the repletion of vitamin D, vitamin D supplementation should be reserved only for the purposes of bone health or other general health needs [18].

## 4. Physical Activity in Cancer Survivors

Physical activity and exercise have beneficial effects on health-related quality of life domains in cancer survivors, including fear of recurrence (e.g., breast cancer), body image/self-esteem, emotional well-being, sexuality, sleep disturbance, social functioning, anxiety, fatigue, and pain [78,79]. The benefits of physical activity and exercise are further strengthened by results of observational studies reporting that regular physical activity (3–5 days/week for at least 30 min per session, 50%–70% of heart rate reserve) is associated with reduction in cancer-specific mortality and all-cause mortality in early-stage breast [80,81], prostate [82] and colorectal cancer [83,84,85,86,87] Although nearly 66% of survivors do not meet national physical activity guidelines, those meeting the guidelines report better quality of life in multiple domains compared to less active individuals [88,89].

Moderate to vigorous-intensity activity like brisk walking, bicycling, or swimming for approximately 3 h per week decreased the risk of all-cause and cancer-specific mortality in postmenopausal breast cancer survivors, as shown by the Women’s Health Initiative study [81]. A meta-analysis of six breast cancer studies, including over 10,000 women, suggested that physical activity after diagnosis reduced breast cancer recurrence as well as cancer-specific and all-cause mortality by 24%, 34%, and 41%, respectively. The benefit was more pronounced in females with BMI >25 kg/m^2^ [90]. In colon cancer, a 50% reduction in the risk of recurrence and improved overall survival was seen in stage III survivors enrolled in CALGB 89803 who self-reported engaging in over 18 MET-hours (six or more hours) per week, compared to those engaged in less than three MET-hours (less than 3 h) per week [91]. Similar results were observed in 1339 women with Stage I–III colorectal cancer enrolled on the Women’s Health Initiative study [83]. In the Health Professionals Follow-up Study, the activity level required for male colorectal survivors to achieve significant survival benefit was higher at 27 MET-hours (nine or more hours) per week [84]. Prostate cancer survivors evaluated in this study who participated in at least three hours per week of vigorous activity had a lower risk of all-cause and prostate cancer-specific mortality compared to those engaged in vigorous activity for less than one hour per week (49% and 61%, respectively) [82].

In addition to aerobic physical activity, supervised resistance training may be beneficial in some survivor populations. In the past, breast-cancer survivors with lymphedema or at risk of lymphedema due to axillary lymph node dissection were encouraged to limit the use of their arm and avoid lifting heavy weights due to concerns for lymphedema exacerbation. The Physical Activity in Lymphedema (PAL) study evaluated progressive weight training in breast-cancer survivors with lymphedema and showed a lower incidence of lymphedema exacerbations, reduced symptoms, and increased strength in the intervention group compared to the control group [92]. In those at risk for lymphedema, slowly progressive weight lifting did not increase the incidence of lymphedema when compared to no exercise [92]. Hence, supervised graduated resistance training is safe and potentially beneficial in this group of patients. Importantly, survivors should be evaluated for arm or shoulder morbidity before beginning a new upper body exercise program [93].

Physical activity may also improve treatment-related side effects. For example, weight-bearing exercise, especially resistance training, has a positive effect on bone mineral density as shown in population-based studies of persons without cancer [85]. In a meta-analysis, exercise training programs were found to reverse almost 1% of bone loss per year in the lumbar spine and femoral neck in both pre-and postmenopausal women [86]. Though not well studied in cancer survivors, these benefits may be especially relevant in survivors on endocrine therapies that increase risk of osteoporosis [87]. Aromatase inhibitor (AI) induced arthralgia is a major reason for poor compliance with adjuvant endocrine therapy in breast cancer survivors. A recent prospective study demonstrated that exercise led to improvement in AI-induced arthralgia in the inactive breast cancer survivors, offering a low-cost means to potentially increase compliance in this population [94]. Routine physical activity may also be helpful in addressing chronic fatigue during and after cancer therapy. A recent prospective study of breast cancer patients engaging in various levels of physical activity during and after chemotherapy demonstrated lower rates of fatigue during chemotherapy with engagement in at least a moderate level of physical activity, when compared to those assigned to usual care [95]. A study in colorectal cancer survivors also supports the benefits of physical activity in lowering the impact of fatigue, with those survivors meeting national physical activity guidelines reporting lower fatigue scores than their more sedentary counterparts [96]. In addition, two meta-analyses of multiple studies evaluating the impact of physical activity on cancer-related and chemotherapy- or radiation-related fatigue demonstrated the positive effect of regular participation in physical activity [97,98].

Various mechanisms have been proposed to explain the protective benefit of physical activity and exercise in cancer patients. Routine physical activity may produce alterations in immune function, oxidative damage, and the insulin axis that may impact cancer metabolism. Exercise training exerts anti-inflammatory and anti-proliferative effects by decreasing levels of factors like cyclooxygenase-2 (COX-2), inducible nitric oxide synthase, and TNF-α, all of which play a role in tumorigenesis [99,100,101]. Given the reductions in cancer-specific and overall health outcomes seen in cohort studies, a number of randomized physical activity intervention studies and cohort studies are currently underway in breast, colon, and prostate cancer survivors. Many of these include correlative studies evaluating biologic mechanisms for the benefit of physical activity and quality of life. These studies are testing a variety of structured physical activity programs on cancer-specific and health-related outcomes, with results eagerly awaited [40,102,103].

There are several barriers to physical activity in cancer survivors, some related to previous cancer therapy. Permanent sensory neuropathy associated with oxaliplatin has been reported in 15%–40% of colon cancer survivors, up to 6 years after completion of adjuvant therapy [104,105]. Similarly, it has been shown that all forms of lung cancer therapies lead to varying degrees of physical or functional impairments that can dramatically reduce a patient’s ability to tolerate exercise [106]. Nearly 90% of all cancer patients experience pain during the course of their illness [107] and about 20%–30% have cancer- or treatment-related chronic pain [108,109]. Physicians are also less likely to recommend exercise for cancer survivors regardless of weight [60]. This may be in part related to the lack of clear evidence delineating the optimal type, intensity, frequency and duration of physical activity necessary to improve cancer- or treatment-related outcomes in a given cancer type during a specific phase of survivorship. Apart from lack of guidelines to help patients, physicians are also pressed for time to allow for a detailed discussion about healthy lifestyle and exercise benefits/options [61,110,111,112]. These issues hinder widespread acceptance of exercise and physical activity in cancer survivors and highlight areas for future research.

Cancer survivors without major physical restrictions should be advised to avoid inactivity and counseled on the standard guidelines endorsed by the ACS, NCCN, and American College of Sports Medicine [48,77,113]. Those embarking on a new program of physical activity or who are deemed to be at moderate to high-risk for adverse events with exercise (*i.e.*, survivors with peripheral neuropathy, lymphedema, ostomy, lung or abdominal surgery, or cardiovascular or musculoskeletal comorbidities) should consider working with a trained rehabilitation or exercise specialist, if available [48]. Guideline recommendations include participating in at least 150 min of moderate intensity or 75 min of vigorous intensity activity each week, preferably spread throughout the week. Additionally, two or three weekly resistance or strength training sessions involving major muscle groups should be encouraged. Stretching of major muscle groups should be performed on the days of other exercise [48]. As most survivors are not meeting guideline recommendations, short and long-term goals for physical activity should be set, including incremental increases in physical activity engagement and variation of types of activity [48].

## 5. Smoking Cessation and Survivors

The benefits of smoking cessation in the general population, and in cancer survivors specifically, are well described. Engaging in smoking behaviors can have negative consequences on cancer outcomes. For example, a meta-analysis of ten studies of lung cancer survivors demonstrated that continued smoking after diagnosis was associated with a significantly increased risk in all-cause mortality and recurrence in early stage disease [114]. Estimated five year survival rates for smokers and non-smokers or former smokers were 33% and 70%, respectively [114]. A history of ever-smoking can also impact outcomes in colon cancer, as a recent analysis of the adjuvant N0147 study demonstrated a significantly shorter disease-free survival in ever-smokers [114,115]. Similar findings have emerged in head and neck and bladder cancer [116,117]. Although most cancer survivors do not engage in smoking behaviors after treatment, approximately 15% of cancer survivors continue to smoke after diagnosis [59]. Distress associated with cancer diagnosis has been associated lack of engagement in health behaviors like exercise and smoking cessation [118,119].

Realizing that recommendations from health care providers have a powerful impact on patient behavior [120], a growing proportion of survivors could derive benefits from provider-driven recommendations and interventions designed to promote smoking cessation. Survivors should be assessed for tobacco use at each visit, with encouragement for cessation at all available opportunities [121]. Various methods to facilitate cessation may range from counseling to pharmacotherapy. Current U.S. Public Health Service Clinical Practice guidelines for smoking cessation recommend employing the 5A’s (Ask, Advise, Assess, Assist and Arrange) and recommend combination therapy involving behavioral and pharmacologic interventions, as this has been shown to be more effective than any component alone [122]. FDA approved medications include nicotine replacement, sustained release bupropion and vereniciline. National hotlines and community-based programs are also important resources for survivors interested in smoking cessation. As smoking cessation may improve both cancer- and overall health-related outcomes, the NCCN Survivorship guidelines recommend counseling about smoking cessation for all cancer survivors as part of routine survivorship care [123] and specific clinical practice guidelines in smoking cessation were released by the NCCN at their 2015 Annual Conference. ASCO has also released a guide to help providers and patients work in a stepwise fashion to quit smoking [124,125], while ACS has a guide to quitting smoking available on their website [126].

## 6. Conclusions

Increasing evidence is highlighting the role of lifestyle behaviors in influencing cancer-related outcomes, ranging from quality of life to prognosis. Obesity, poor dietary choices, inactivity, and continued smoking have repeatedly been shown to negatively impact outcomes in cancer survivors of most common cancer types as shown by many observational and a few interventional studies. Interventional studies like WINS and WHEL have demonstrated the feasibility of large-scale lifestyle modification interventions in survivors, but the optimal type and duration of physical activity in specific cancer populations has not been well-defined. While ongoing interventional studies will evaluate large-scale implementation of lifestyle modifications in a variety of cancer settings and are eagerly awaited, current guideline recommendations from professional organizations such as the American Cancer Society, American Society of Clinical Oncology, and the National Comprehensive Cancer Network are worth discussing with most survivors and those undergoing cancer therapy. In addition, evaluation of the biologic mechanisms underlying how lifestyle modifications and health behaviors affect cancer outcomes is needed.

Recognizing a cancer diagnosis as a teachable moment, receptivity of lifestyle recommendations is high in survivors if physicians, as authority figures, educate survivors and endorse these recommendations. Hence lifestyle recommendations form an important part of survivorship care and should in included in the survivorship care planning and delivery. While we await data from large-scale health behavior intervention studies, survivor and provider education of generalized healthy lifestyle guidelines and recommendations are worthwhile and may impact long-term health outcomes significantly.

## Appendix

**Table A1:** Survivorship Guidelines: Healthy Behavior and Lifestyle Factors.

	Weight Management	Nutrition	Physical Activity	Smoking Cessation
NCCN [48]	Achieve and maintain a healthy body weight throughout life Pay attention to calories consumed *vs.* calories expended Achieve and maintain normal BMI Weigh oneself weekly to monitor weight loss/gain	Maintain a healthy diet high in fruits, vegetables and whole grains and low in red/processed meat, sugars and fats to help promote healthy weight and avoid obesity Minimize alcohol intake Limit to one drink per day for women and two per day for men	Engage in physical activity regularlya. Avoid inactivity, engage in general physical activity daily (taking the stairs, parking in the back of parking lot) Strive for at least 150 min of moderate or 75 min of vigorous activity per week (spread out over the week)	Avoid tobacco products
ACS [18]	Achieve and maintain a healthy weight. If overweight or obese, limit consumption of high-calorie foods and beverages and increase physical activity to promote weight loss.	Achieve a dietary pattern that is high in vegetables, fruits, and whole grains. Follow the American Cancer Society Guidelines on Nutrition and for Cancer Prevention.	Engage in regular physical activity. Avoid inactivity and return to normal daily activities as soon as possible following diagnosis. Aim to exercise at least 150 min per week. Include strength training exercises at least 2 days per week.	Avoid tobacco
ASCO [124]	Tool for clinicians and providers to manage obesity Education and awareness about energy balance and cancer risk association Provides resources to help tackle obesity.	Endorses ACS guidelines	Endorses ACS guidelines	Recommend organizing a patient’s quit attempt around the “5 As”—Ask, Advise, Assess, Assist, and Arrange
AICR [42]	Be as lean as possible without becoming underweight	Avoid sugary drinks. Limit consumption of energy-dense foods. Eat more of a variety of vegetables, fruits, whole grains and legumes such as beans. Limit consumption of red meats (such as beef, pork and lamb) and avoid processed meats. If consumed at all, limit alcoholic drinks to 2 for men and 1 for women a day. Limit consumption of salty foods and foods processed with salt (sodium). Don’t use supplements to protect against cancer.	Be physically active for at least 30 min every day. Limit sedentary habits.	Do not smoke or chew tobacco
ACSM [93]	No recommendations	No recommendations	Exercise is safe during and after cancer treatment, and should be encouraged. General medical assessment before embarking on new physical activity program Follow Physical activity guidelines for Americans (150 min per week) Adapt exercise /physical activity program to accommodate disease and treatment-related late/long-term effects.	No recommendations

NCCN: National Comprehensive Cancer Network; ASCO: American Society of Clinical Oncology; AICR: American Institute of Cancer Research; ACS: American Cancer Society; ACSM: American College of Sports Medicine.
